# Supplementary material for: Acetazolamide modulates intracranial pressure directly by its action on the cerebrospinal fluid secretion apparatus
Source: Fluids Barriers CNS. 2022 Jun 29;19:53. doi: 10.1186/s12987-022-00348-6 (PMC9245291; doi:10.1186/s12987-022-00348-6)
Supplement: Supplementary file 1 — Additional file 1. Blood gas analysis and telemetric ICP measurements. [file 12987_2022_348_MOESM1_ESM.pdf]

	CONTROL									ACETAZOLAMIDE									2way ANOVA p-value	
	Baseline			1h			2h			Baseline			1h			2h				
	Average	SD	n	Average	SD	n	Average	SD	n	Average	SD	n	Average	SD	n	Average	SD	n	Time	Treatment
pH	7.393	0.020	5	7.359	0.019	5	7.337	0.031	5	7.379	0.024	5	7.156	0.027	5	7.126	0.019	5	<.001	<.001
pCO_2	5.26	0.31	5	5.15	0.27	5	5.38	0.30	5	5.21	0.35	5	9.40	0.34	5	10.05	0.40	5	<.001	<.001
pO_2	13.8	1.1	5	13.8	1.3	5	14.0	1.4	5	14.4	3.0	5	15.5	1.8	5	14.5	1.4	5	0.215	0.402
ctHb	9.5	0.3	5	9.2	0.4	5	9.6	0.3	5	9.3	0.4	5	9.2	0.4	5	9.5	0.7	5	0.033	0.729
sO_2	94.2	1.7	5	94.7	3.7	5	93.3	2.2	5	94.3	2.9	4	90.2	3.1	5	88.6	3.0	5	<.001	0.151
FO_2Hb	94.5	1.8	5	94.1	2.7	5	93.3	2.4	5	95.3	4.1	5	90.1	3.3	5	88.5	3.2	5	<.001	0.181
K^+	5.2	0.2	5	4.9	0.2	5	5.0	0.3	5	5.4	0.4	5	5.1	0.4	5	5.9	0.6	5	0.038	0.043
Na^+	139	1.3	5	142	1.6	5	144	2.4	5	138	1.7	5	143	2.2	5	144	2.9	5	<.001	0.77
Ca^{2+}	0.87	0.05	5	0.84	0.03	5	0.85	0.05	5	0.79	0.17	5	0.78	0.14	5	0.82	0.09	5	0.28	0.389
Cl^-	102	1.6	5	107	2.0	5	110	1.1	5	101	2.1	5	105	1.5	5	106	1.6	5	<.001	0.047
cGlu	22.6	1.7	5	18.4	2.6	5	11.9	2.2	5	24.5	1.4	5	19.5	1.9	5	14.4	2.0	5	<.001	0.09
cLac	1.2	0.4	5	1.6	0.4	5	2.0	0.5	5	1.1	0.3	5	0.8	0.2	5	0.9	0.2	5	0.223	<.001
ctBil	53	5.9	5	48	4.0	4	54	8.6	5	50	2.6	3	39	2.6	3	42	3.1	3	0.001	0.09
cBase	-0.9	1.1	5	-3.7	1.2	5	-4.2	1.6	5	-2.2	1.3	5	-3.9	1.3	5	-4.4	1.2	5	<.001	0.482
$cHCO_3^-$	23.7	0.8	5	21.6	0.9	5	21.1	1.3	5	23.0	0.9	4	19.6	1.1	4	19.3	0.7	4	<.001	0.04

Supplementary Table S1 Blood gas analysis in intact animals before (baseline) and after (at 1 and 2 h) i.v. injection of 100 mg kg^{-1} AZE or control solution in anesthetized and ventilated rats.

	CONTROL NEPHRECTOMY									ACETAZOLAMIDE NEPHRECTOMY									2way ANOVA p-value	
	Baseline			1h			2h			Baseline			1h			2h				
	Average	SD	n	Average	SD	n	Average	SD	n	Average	SD	n	Average	SD	n	Average	SD	n	Time	Treatment
pH	7.343	0.031	4	7.295	0.039	4	7.264	0.024	4	7.368	0.036	4	7.148	0.027	4	7.100	0.035	4	<.001	0.005
pCO_2	5.34	0.34	4	4.78	0.52	4	5.41	0.19	4	4.99	0.16	4	9.08	0.42	4	10.16	0.58	4	<.001	<.001
pO_2	16.4	1.2	4	16.4	1.4	4	14.5	3.4	4	16.3	3.1	4	17.1	1.4	4	16.5	2.1	4	0.303	0.539
ctHb	9.4	0.2	3	9.0	0.4	4	9.0	0.3	4	9.6	1.0	4	9.2	0.7	4	8.7	0.4	4	0.136	0.761
sO_2	97.2	3.9	3	96.3	3.7	4	93.0	6.9	4	96.4	4.0	4	94.5	2.6	4	91.2	2.6	4	0.033	0.719
FO_2Hb	96.9	3.0	3	95.6	2.3	4	92.2	5.9	4	95.7	2.9	4	94.8	3.1	4	91.9	1.9	3	0.059	0.819
K^+	5.8	0.8	4	5.1	0.2	4	6.0	0.4	4	5.6	0.1	4	5.7	0.3	4	6.2	0.4	4	0.025	0.382
Na^+	138	1.5	4	143	1.3	4	144	0.8	4	139	2.2	4	143	0.5	4	145	0.8	4	<.001	0.072
Ca^{2+}	0.84	0.11	4	0.82	0.08	4	0.85	0.12	4	0.84	0.04	4	0.71	0.10	4	0.77	0.10	4	0.033	0.321
Cl^-	102	3.7	4	108	1.7	4	109	1.5	4	102	1.0	4	105	1.9	4	106	1.9	4	<.001	0.062
cGlu	24.3	5.6	4	14.7	3.3	4	11.0	2.6	4	24.8	3.2	4	20.4	3.3	4	14.8	1.9	4	<.001	0.132
cLac	1.4	0.4	4	1.9	0.2	4	1.9	0.5	4	1.0	0.2	4	0.7	0.2	4	0.8	0.2	4	0.344	0.003
ctBil	54	2.8	2	45	0.6	3	41	5.8	3	56	11.4	3	42	6.4	2	40	2.1	2		
cBase	-4.0	1.3	4	-9.2	1.3	4	-8.7	1.4	4	-3.8	2.1	4	-5.3	0.9	4	-6.1	1.5	4	<.001	0.043
$cHCO_3^-$	21.4	1.0	4	17.8	1.0	4	17.8	1.0	4	21.7	1.7	4	19.0	0.8	4	18.2	1.1	4	<.001	0.424

Supplementary Table S2 Blood gas analysis before (baseline) and after (at 1 and 2 h) i.v. injection of 100 mg kg^{-1} AZE or control solution in anesthetized, ventilated and nephrectomized rats

	CONTROL HYPERVENTILATION									ACETAZOLAMIDE HYPERVENTILATION									2way ANOVA p-value	
	Baseline			1h			2h			Baseline			1h			2h				
	Average	SD	n	Average	SD	n	Average	SD	n	Average	SD	n	Average	SD	n	Average	SD	n	Time	Treatment
pH	7.434	0.021	4	7.585	0.050	4	7.525	0.083	4	7.425	0.023	4	7.216	0.036	4	7.150	0.050	4	0.001	<.001
pCO_2	4.77	0.22	4	2.04	0.12	4	1.92	0.10	4	4.84	0.19	4	7.80	0.29	4	8.37	0.65	4	0.039	<.001
pO_2	14.4	2.4	4	22.8	0.6	4	22.8	0.6	4	13.8	1.1	4	23.0	0.5	4	23.1	0.7	4	<.001	0.931
ctHb	9.3	0.3	4	9.3	0.7	4	9.8	0.7	4	9.8	0.4	4	9.7	0.4	4	10.4	0.5	4	0.022	0.128
sO_2	95.3	1.4	4	NA			NA			95.6	3.2	4	97.7	2.4	4	97.3	2.7	4		
FO_2Hb	95.5	1.4	4	NA			NA			95.0	2.0	4	97.4	1.3	4	97.2	1.6	4		
K^+	5.2	0.2	4	5.2	0.2	4	4.9	0.2	4	5.4	0.5	4	5.8	0.5	4	6.2	0.4	4	0.185	0.009
Na^+	139	1.7	4	140	1.0	4	142	3.5	4	139	0.0	4	142	0.8	4	143	1.6	4	0.007	0.326
Ca^{2+}	0.92	0.04	4	0.88	0.03	4	0.86	0.07	4	0.87	0.05	4	0.86	0.01	4	0.82	0.03	4	0.094	0.056
Cl^-	102	2.2	4	106	2.2	4	110	3.2	4	102	1.0	4	105	1.7	4	108	1.3	4	<.001	0.377
cGlu	19.5	2.9	4	21.7	3.1	4	16.0	3.7	4	22.7	2.1	4	21.9	3.9	4	19.5	4.4	4	0.001	0.352
cLac	1.1	0.2	4	4.2	1.0	4	4.6	0.7	4	1.1	0.2	4	0.8	0.2	4	1.0	0.2	4	<.001	<.001
ctBil	51	3.1	4	NA			NA			53	7.2	3	53	8.5	2	63	5.7	2		
cBase	-0.3	2.4	4	-7.3	2.5	4	-10.8	3.6	4	-0.7	1.1	4	-4.1	1.7	4	-7.1	1.7	4	<.001	0.205
$cHCO_3^-$	24.5	1.8	4	NA			NA			24.1	0.9	4	20.3	1.3	4	17.9	1.4	4		

Supplementary Table S3 Blood gas analysis before (baseline) and after (at 1 and 2 h) i.v. injection of 100 mg kg^{-1} AZE or control solution in anesthetized and hyperventilated rats. NA – the values were over the detection threshold.

	CONTROL INTRAVENTRICULAR									ACETAZOLAMIDE INTRAVENTRICULAR									2way ANOVA p-value	
	Baseline			1h			2h			Baseline			1h			2h				
	Average	SD	n	Average	SD	n	Average	SD	n	Average	SD	n	Average	SD	n	Average	SD	n	Time	Treatment
pH	7.409	0.029	4	7.367	0.034	4	7.357	0.041	4	7.421	0.019	4	7.387	0.005	4	7.358	0.014	4	<.001	0.526
pCO_2	4.76	0.36	4	4.47	0.25	4	4.76	0.29	4	4.77	0.15	4	4.70	0.30	4	4.78	0.20	4	0.23	0.551
pO_2	16.3	3.3	4	15.8	1.4	4	14.9	1.3	4	15.2	2.7	4	14.9	1.9	4	15.0	1.7	4	0.388	0.649
ctHb	9.3	0.5	4	9.5	0.5	4	9.6	0.5	4	9.5	0.6	4	9.9	0.3	4	9.9	0.3	4	0.167	0.289
sO_2	95.6	1.4	4	95.2	1.0	4	94.4	2.2	4	95.0	1.9	4	94.0	1.6	4	93.9	1.4	4	0.089	0.487
FO_2Hb	96.0	1.5	4	95.3	1.1	4	94.6	2.2	4	95.1	2.1	4	94.2	1.6	4	94.1	1.4	4	0.074	0.487
K^+	5.2	0.4	4	4.8	0.5	4	5.0	0.4	4	5.2	0.2	4	5.0	0.3	4	5.2	0.7	4	0.263	0.582
Na^+	140	0.5	4	144	2.1	4	144	0.8	4	141	2.1	4	143	2.2	4	145	0.0	4	0.007	0.55
Ca^{2+}	0.88	0.11	4	0.85	0.08	4	0.85	0.06	4	0.89	0.05	4	0.86	0.06	4	0.86	0.06	4	0.081	0.895
Cl^-	104	3.7	4	110	3.1	4	108	4.7	4	101	2.5	4	106	1.3	4	109	3.4	4	0.024	0.204
cGlu	19.8	2.6	4	13.1	1.2	4	8.4	1.6	4	21.4	3.4	4	17.5	2.2	4	9.9	0.8	4	<.001	0.081
cLac	1.5	0.2	4	2.3	0.5	4	1.9	0.4	4	1.1	0.2	4	1.9	0.4	4	2.0	0.3	4	0.001	0.313
ctBil	51	5.1	4	51	5.5	4	55	3.8	4	52	6.1	4	57	3.7	4	59	3.6	4	<.001	0.284
cBase	-2.1	3.4	4	-6.1	2.3	4	-5.5	2.3	4	-1.3	0.7	4	-3.8	1.1	4	-5.4	1.7	4	0.001	0.447
$cHCO_3^-$	23.1	2.4	4	19.5	2.1	4	18.2	4.2	4	23.7	0.6	4	21.8	0.6	4	20.6	1.1	4	0.001	0.265

Supplementary Table S4 Blood gas analysis before (baseline) and after (at 1 and 2 h) the i.c.v. infusion of 18 mM AZE (expected ventricular concentration 500 µM) or control solution in anesthetized and ventilated rats.

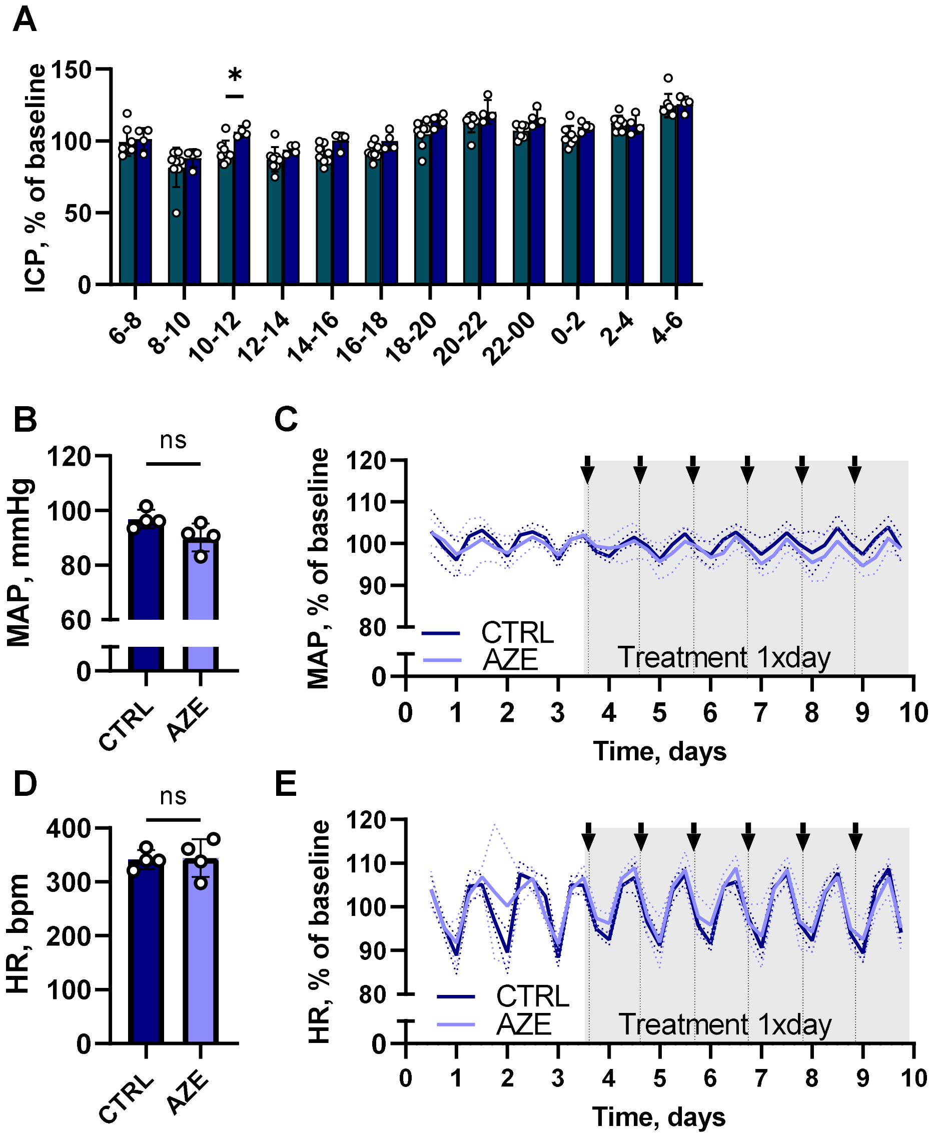

Supplementary Figure 1 Telemetric measurements in awake rats. Comparison between the baseline daily ICP fluctuations and p.o. saline treatment is shown in **A.** Data is presented as 2 h average ICP normalized to the average 24 h ICP before the treatment, and presented for baseline period (green, n=8) and for 1 × day p.o. saline (dark blue, n = 4). 2way ANOVA with Bonferroni's multiple comparisons test only showed significance 2h after the p.o. drug administration (P = 0.021). Figure **B** represents the 72 h average MAP, separated between the animals that afterwards received either 1 × daily 100 mg kg-1 p.o. AZE treatment or control (MAP_{CTRL} = 97 ± 3 mmHg, MAP_{AZE} = 90 ± 5 mmHg, n = 4 in each group, P = 0.07). The daily MAP fluctuations, normalized to the 24h baseline MAP are shown as 6 h bins in C. The heart rate data is shown in D (HR_{CTRL} = 342 ± 17 bpm, HR_{AZE} = 344 ± 35 bpm, n = 4 in each group, P = 0.9) and E in the same manner as the MAP. Data are shown as mean ± SD. *; P < 0.05, ns; not significant.
